# Development and Application of a Simple “Easy To Operate” Propidium Monoazide-Crossing Priming Amplification on Detection of Viable and Viable But Non-culturable Cells of O157 *Escherichia coli*

**DOI:** 10.3389/fmicb.2020.569105

**Published:** 2020-09-25

**Authors:** Wenqu Zhou, Kan Wang, Wei Hong, Caiying Bai, Ling Chen, Xin Fu, Tengyi Huang, Junyan Liu

**Affiliations:** ^1^GMU-GIBH Joint School of Life Sciences, Guangzhou Medical University, Guangzhou, China; ^2^Research Center for Translational Medicine, The Second Affiliated Hospital, Shantou University Medical College, Shantou, China; ^3^Guangdong Women and Children Hospital, Guangzhou, China; ^4^School of Food Science and Engineering, Guangdong Province Key Laboratory for Green Processing of Natural Products and Product Safety, South China University of Technology, Guangzhou, China; ^5^Research Institute for Food Nutrition and Human Health, Guangzhou, China; ^6^Department of Laboratory Medicine, The Second Affiliated Hospital of Shantou University Medical College, Shantou, China; ^7^Department of Civil and Environmental Engineering, University of Maryland, College Park, MD, United States

**Keywords:** propidium monoazide-crossing priming amplification, viable but non-culturable state, *Escherichia coli* O157:H7, rapid detection, foodborne pathogens

## Abstract

O157 *Escherichia coli* is one of the most important foodborne pathogens causing disease even at low cellular numbers. Thus, the early and accurate detection of this pathogen is important. However, due to the formation of viable but non-culturable (VBNC) status, the golden standard culturing methodology fails to identify O157 *E. coli* once it enters VBNC status. Crossing priming amplification (CPA) is a novel, simple, easy-to-operate detection technology that amplifies DNA with high speed, efficiency, and specificity under isothermal conditions. The objective of this study was to firstly develop and apply a CPA assay with propidium monoazide (PMA) for the rapid detection of the foodborne *E. coli* O157:H7 in VBNC state. Five primers (2a/1s, 2a, 3a, 4s, and 5a) were specially designed for recognizing three targets, which were *rfbE*, *stx1*, and *stx2*, and evaluated for its effectiveness in detecting VBNC cell of *E. coli* O157:H7 with detection limits of pure VBNC culture at 10^3^, 10^5^, and 10^5^ colony-forming units (CFUs)/ml for *rfbE*, *stx1*, and *stx2*, respectively, whereas those of food samples (frozen pastry and steamed bread) were 10^3^, 10^5^, and 10^5^ CFUs/ml. The application of the PMA-CPA assay was successfully used on detecting *E. coli* O157:H7 in VBNC state from food samples. In conclusion, this is the first development of PMA-CPA assay on the detection of VBNC cell, which was found to be useful and a powerful tool for the rapid detection of *E. coli* O157:H7 in VBNC state. Undoubtedly, the PMA-CPA method can be of high value to the food industry owing to its various advantages such as speed, specificity, sensitivity, and cost-effectiveness.

## Highlights

- This is the first development of a PMA-CPA method on VBNC detection.
- This detection assay has been successfully applied to three targets of *Escherichia coli* in VBNC state.
- This study proposes the necessity for viable cell detection instead of the conventional “golden standard” culturing methodology.

## Introduction

In recent years, the outbreak of foodborne diseases caused by pathogens and its related virulence factors is a major threat for many countries, although much attention had been paid to food safety issues. Foodborne pathogens including enterohemorrhagic *Escherichia coli* (EHEC), *Staphylococcus aureus*, *Vibrio parahaemolyticus*, and *Pseudomonas aeruginosa* have posed great concern in the food industry and public health (Miao et al., 2016, 2019; Xie et al., 2017a; Xu et al., 2019), especially their acquisition of antimicrobial resistance (Xu et al., 2007, 2011a, 2017b; Yu et al., 2016; Xie et al., 2017b; Liu et al., 2018c,d), biofilm formation (Lin et al., 2016; Bao et al., 2017a; Miao et al., 2017b), and toxin production (Liu et al., 2016a). EHEC causes more than 63,000 illnesses, 2,100 hospitalizations, and 20 deaths each year in the United States (Jiang et al., 2016). *E. coli* O157:H7 is the main EHEC serotype that causes the majority of EHEC human infections. As one of the most commonly found foodborne pathogens, it can be transmitted by contaminated food such as cattle, milk, eggs and vegetables, rice cakes, and others (Ackers et al., 1998; Jacob et al., 2013; Marder et al., 2014). The methods are labor-intensive and time-consuming and cannot meet the requirement of rapid monitoring. Hence, developing a rapid, sensitive, and accurate method to detect *E. coli* O157:H7 in various foods with a complex matrix is crucial in preventing disastrous *E. coli* O157:H7 outbreaks and associated human infections.

In 1982, Xu et al. (1982) firstly reported “viable but non-culturable” (VBNC) state, which was considered to be a survival strategy of non-spore-forming bacteria in response to adverse conditions (Xu et al., 1982; Oliver, 2010). Bacteria can enter into VBNC state by the stimulation of adverse environmental conditions, such as low temperature, nutrient-limited conditions, high salt, low pH, and even ultraviolet-induced (Foster, 1999; Ramaiah et al., 2002; Cunningham et al., 2009; Deng et al., 2015; Liu et al., 2017a,b, 2018a,b; Guo et al., 2019). For now, 85 species of bacteria have been confirmed that can enter into VBNC state, including 18 non-pathogenic and 67 pathogenic species (Li et al., 2014). In recent years, many studies have determined that VBNC bacteria still can produce harmful substances (Deng et al., 2015; Liu et al., 2017a,b, 2018a,b). *Shigella dysenteriae* and *E. coli* O157 still retain Shiga toxin encoding gene (*stx*) and produce toxin in VBNC state (Rahman et al., 1996; Liu et al., 2017d). Moreover, bacteria in VBNC can resuscitate and grow again with suitable conditions (Pinto et al., 2015). Nowadays, the traditional culture method and a nucleic acid detection method are widely used. However, the discovery of VBNC in recent years has brought difficulties to the detection method. The VBNC cell cannot grow on the plate medium due to low metabolic activity, which means that normal culture methods can lead to false-negative results of detection. Hence, rapid and accurate identification of VBNC bacteria is of utmost importance. Bacteria in the VBNC state remain metabolically and physiologically viable and continuing to express virulence genes (Liu et al., 2016b, c, d, 2017a, 2017c,2018a; Bao et al., 2017b; Xu et al., 2017a). Concerning conventional detection methods that are time-consuming and only suitable for experimental use, alternative rapid and cost-effective detection methods are desperately required to detect VBNC pathogens (Lin et al., 2016; Xu et al., 2016; Miao et al., 2017a; Zhao et al., 2018). As the ethidium bromide monoazide (EMA) or propidium monoazide (PMA) penetrates only into dead bacterial cells with compromised membrane integrity but not into live cells with intact cell membranes, EMA/PMA treatment to cultures with both viable and dead cells results in selective removal of DNA from dead cells. Therefore, scientists had utilized EMA/PMA combined with nucleic acid amplification techniques to complete the detection of VBNC cells. Recently, nucleic acid amplification methods combined with EMA/PMA have been widely used for the detection of pathogenic bacteria in the VBNC state, such as PMA-polymerase chain reaction (PCR) (Liu Y. et al., 2018; Zhong and Zhao, 2018).

Although numerous quick methods based on nucleic acids, such as PCR (Gehring and Tu, 2005; Xu et al., 2011b) and real-time PCR (O’Hanlon et al., 2004), have been developed and used for detecting *E. coli* O157:H7 in food, a quicker and less expensive technology is always most preferred. Isothermal amplification is a novel method for DNA amplification at a constant temperature, providing simple, fast, independent of sophisticated instruments and cost-effective techniques to detect biological targets, especially for less well-equipped laboratories, as well as for filed detection. Isothermal technologies mainly include loop-mediated isothermal amplification, rolling circle amplification, single primer isothermal amplification, polymerase spiral reaction, strand displacement amplification, and recombinase polymerase amplification (Walker et al., 1992; Notomi et al., 2000; Haible et al., 2006; Zhao et al., 2009, 2010, 2011; Wang et al., 2011; Xu Z. et al., 2012; Xu et al., 2020; Liu et al., 2015; Yang et al., 2017). Cross priming amplification (CPA) is a novel isothermal method that relies on five primers (2a/1s, 2a, 3a, 4s, and 5a) to amply the target nucleotide sequences (Xu G. et al., 2012). It does not require any special instrumentation and presents all the features, including rapidity, specificity, and sensitivity. Recently, CPA assays have been used for the detection of *E. coli* O157:H7, *Listeria monocytogenes*, *Enterobacter sakazakii*, *Salmonella enterica, Yersinia enterocolitica*, and other pathogens (Yulong et al., 2010; Wang et al., 2014, 2018; Zhang et al., 2015; Xu et al., 2020). Introducing the cross-priming principles, CPA is advantageous on reproducibility and stability with a similar level of sensitivity, specificity, and rapidity comparing with loop-mediated isothermal amplification, the most broadly applied isothermal methodologies. Therefore, CPA is a potentially valuable tool for the rapid diagnosis of foodborne pathogens, as well as those in the VBNC state. However, there is no report of PMA-CPA assay for the detection of VBNC *E. coli* O157:H7.

This study aimed to develop a rapid PMA-CPA assay to detect *E. coli* O157:H7 in VBNC state targeting on *rfbE*, *stx1*, and *stx2* genes combining visual methods with the addition of calcein and applying this assay to detect the *E. coli* O157:H7 strains from food samples.

## Materials and Methods

### Induction of Entry Into the Viable But Non-culturable State in Saline and Food Sample

Induction of VBNC cells and the establishment of PMA-CPA assays were performed on *E. coli* O157 ATCC43895. The bacterial strain was incubated in trypticase soy broth (Huankai Microbial, China) to reach the exponential phase [∼10^9^ colony-forming units (CFUs)/ml]. To induce the entry of VBNC state, the culture was diluted to the final density of 10^8^ CFU/ml with saline (pure culture system) and food homogenate (Cantonese rice cake, Guangzhou Restaurant, Guangzhou) (food system) and stored at −20°C.

### Determination of the Culturable and Viable But Non-culturable State of *E. coli* O157

The conventional plate counting method was used to determine the cultivability of *E. coli* O157. The induction culture was serial diluted with 0.9% sodium chloride and inoculated on trypticase soy agar at 37°C for 24 h. When the number of colonies was <1 CFU/ml for 3 days, it was considered that the survived cells might have entered into the non-culturable state (Deng et al., 2015). Also, the final determination of the VBNC cell was evaluated by the LIVE/DEAD BacLight bacterial viability kit (Thermo Fisher Scientific, China) (Liu et al., 2017d). The stained induction culture was observed by fluorescence microscopy.

### Design of Crossing Priming Amplification Primers

As mentioned before (Xu et al., 2020), the CPA primers were designed for specific O-antigen *rfbE* gene and Shiga toxin genes *stx1* and *stx2* of *E. coli* via Primer Premier 5. For each of the target genes, a set of primers were designed, including five primers that recognized five distinct regions on corresponding sequences. Primers used in this study have been enumerated in Tables 1, 2. All primers were assessed for specificity before use in CPA assays by doing a Blast search with a sequence in GenBank^1^.

**TABLE 1:** Reference strains and results of CPA assays.

Reference strains		PCR assays			CPA results		

Gram-negative organisms	No. of strains	*rfbE*	*stx1*	*stx2*	*rfbE*	*stx1*	*stx2*
*Escherichia coli* O157:H7 ATCC43895	1	+	+	+	+	+	+

**TABLE 2:** Primers sequence for detection.

**Target gene**	**Primers**	**Sequence (5′-3′)**
*rfbE*	4s	AGGACCGCAGAGGAAAGA
	5a	TCCACGCCAACCAAGATC
	2a/1s	AGTACATTGGCATCGTGTCAGATAAACTCATCGAAACA
	2a	AGTACATTGGCATCGTGT
	3a	GGCATCGTGTGGACAGGGT
*stx1*	4s	AGTTGATGTCAGAGGGATAG
	5a	CGCTGTTGTACCTGGAAA
	2a/1s	ATCAGCAAAGCGATAAAACTACGGCTTATTGTTGAA
	2a	ATCAGCAAAGCGATAAAA
	3a	CCTGTTAACAAATCCTGTCAC
*stx2*	4s	GTTACGGGAAGGAATCAGG
	5a	AAATCAGCCACCCACAGC
	2a/1s	CGAACTGACGGTTTACGCATGGGACTTGCCGGTGTT
	2a	CGAACTGACGGTTTACGC
	3a	TGGTCGTACGGACCTTTT

### Development of Propidium Monoazide-Crossing Priming Amplification Assay in Pure Bacterial Culture and Food Sample

For the pure bacterial culture, 500 μl of 10–10^6^-CFU/ml VBNC culture was transformed into 1.5-ml centrifuge tubes. For the food sample culture, the 10–10^6^-CFU/ml cultures were washed three times by saline to avoid the effect of substances in a food sample and placed in 1.5-ml centrifuge tubes. Then, PMA reagent was added to the final concentration of 5 μg/ml. Subsequently, the detection samples mixed with PMA were incubated in the dark at room temperature for 10 min before the tubes were placed horizontally on ice exposed to a halogen lamp (650 W) at a distance of 15 cm for 15 min to complete the combination of DNA and PMA (Chen et al., 2020). The mixed samples were centrifuged at 10,000 r/min for 5 min, and the precipitation under the tubes was processed by DNA extraction kit (Dongsheng Biotech, Guangzhou) followed the instruction of the manufacturer, which were prepared as DNA samples for PMA-CPA.

The CPA reaction was performed as mentioned before (Xu et al., 2020), using thermostatic equipment or water bath in 26 μl, which contained 20-mM Tris–HCl, 10-mM (NH_4_)_2_SO_4_, 10-mM KCl, 8.0-mM MgSO_4_, 0.1% Tween 20, 0.7-M betaine (Sigma), 1.4-mM dNTP (each), 8-U Bst DNA polymerase (NEB, United States), a 1.0-μM primer of 2a/1s, a 0.5-μM (each) primer of 2a and 3a, 0.6-μM (each) primer of 4s and 5a, 1-μl mixture chromogenic agent (mixture with calcein and Mn^2+^), and 1-μl template DNA, and the volume was made up to 26 μl with nuclease-free water. The mixed chromogenic agent consists of 0.13-mM calcein and 15.6-mM MnCl_2_.4H_2_O.

The mixed reaction solution was incubated at 65°C for 60 min and heated at 80°C for 2 min to terminate it. PMA-CPA amplified products were visualized under visible light or the appearance of the laddering pattern on 1.5% agarose gel electrophoresis. However, instead of the laddering pattern, apparent bright strip might present, which can also be regarded as positive results. This experiment was performed in triplicate to ensure reproducibility.

## Results

### Observation of *E. coli* O157 in Viable But Non-culturable State With Fluorescence Microscopy

**FIGURE 1:**
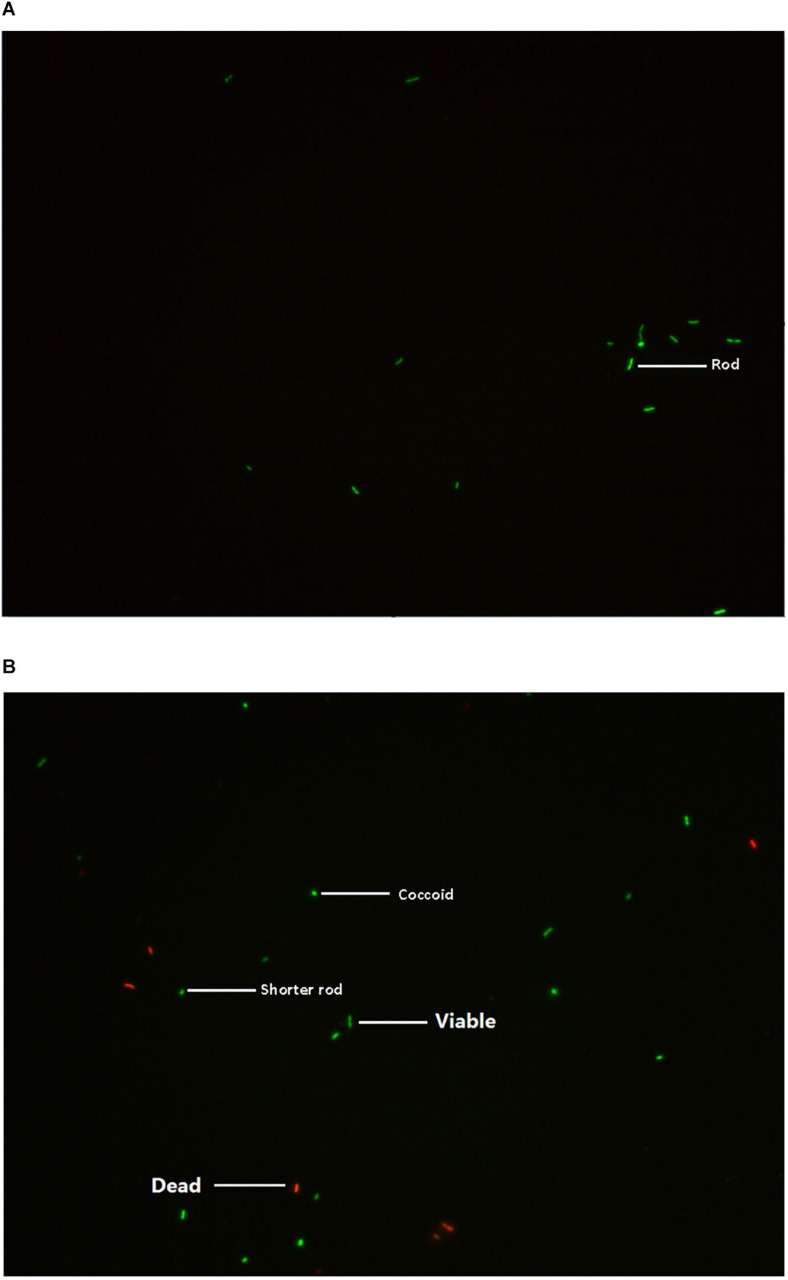
Characterization of *E. coli* O157 in normal viable **(A)** and VBNC state **(B)** under fluorescence microscopy.

The *E. coli* O157 in normal viable and VBNC state was analyzed using the LIVE/DEAD^®^ BacLight Bacterial Viability Kit. Under a fluorescence microscope, the VBNC cells showed green, whereas dead cells exhibited red (Figure 1). The results showed that the VBNC cell might change their morphological characterization from rod-shaped (normal state) to shorter rods or coccoid (VBNC state) (Liu et al., 2017c).

### Propidium Monoazide-Crossing Priming Amplification Assay for Detection of Viable But Non-culturable Cells of *E. coli* O157 in Food Samples

Serial diluted DNA of VBNC cells evaluated the sensitivity of PMA-CPA. There was an obvious color change at the 10^3^–10^6^ CFU/ml DNA, and the ladder-like pattern was clearly observed under ultraviolet light. The detection limits of *E. coli* O157 VBNC were 10^3^, 10^5^, and 10^5^ CFU/ml for *rfbE*, *stx1*, and *stx2* genes, respectively.

**FIGURE 2:**
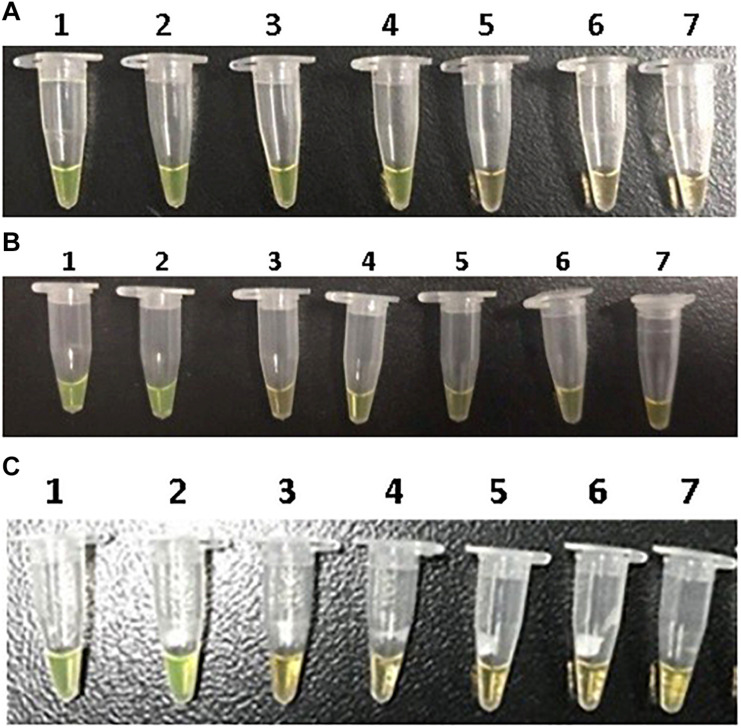
Sensitivity of PMA-CPA assay for detection of VBNC cells of *rfbE* **(A)**, *stx1* **(B)**, and *stx2* **(C)** genes by mixed chromogenic agent in food samples. M-DNA marker; lanes 1–7, 10^6^, 10^5^, 10^4^, 10^3^, 10^2^, and 10 CFU/ml, negative control.

The PMA-CPA assays for the detection of *E. coli* O157 VBNC in food samples were successfully conducted. The detection limits of VBNC cells in the food system were 10^3^, 10^5^, and 10^5^ CFU/ml for *rfbE*, *stx1*, and *stx2* genes, respectively, which were the same as the limits in pure VBNC cells (Figure 2).

## Discussion

*Escherichia coli* O157:H7 is currently a widespread foodborne pathogen throughout the world and has promoted a heightened interest and concern for the low-level detection of these foodborne pathogens (Pennington, 2010). There is a fast-increasing and urgent demand for high-performance techniques for monitoring bacteria in complex foods to reduce the risk of the associated food poisoning. In evaluating detection methodologies for ecologic and epidemiological purposes, a series of attributes should be considered and assessed, including specificity, sensitivity, simplicity, expense, and time. In this study, PMA-CPA assay targeting *rfbE, stx1*, and *stx2* successfully detected detection *E. coli* O157:H7 VBNC cell in pure culture and food samples. Notably, Shiga toxins 1 and 2 (Stx1 and Stx2) encoded by *stx1* and *stx2* can result in gastrointestinal symptoms, such as diarrhea and hemorrhagic colitis, and may also progress to a hemolytic uremic syndrome, a severe sequela of this infection (Saeedi et al., 2017). Considered to be an important health risk in the food testing, the detection of Shiga toxin, especially rapid and easy operating detection assay, may be of utmost significance and urgent necessity. Therefore, we established a PMA-CPA method for the detection of *E. coli* O157:H7 in VBNC cell, as well as its virulence factors. The detection limits of PMA-CPA assay showed consistency with that of CPA assay, no matter in pure bacterial culture or food samples, which had been performed previously (Xu et al., 2020). To the best of our knowledge, this is the first report of a PMA-CPA assay to detect *E. coli* O157:H7 in VBNC state from food samples.

## Conclusion

In conclusion, the designed CPA primers targeted the *rfbE*, *stx1*, and *stx2* genes for the effective detection of *E. coli* O157:H7 VBNC cell. Therefore, being simple, rapid, sensitive, and specific, PMA-CPA assay can be a useful and powerful method in the field and also an alternative diagnostic tool for the detection of *E. coli* O157:H7 in VBNC cell and its related virulence factors in testing as part of an outbreak investigation.

## Data Availability Statement

All datasets presented in this study are included in the article/supplementary material.

## Author Contributions

All authors listed have made a substantial, direct and intellectual contribution to the work, and approved it for publication.

## Conflict of Interest

The authors declare that the research was conducted in the absence of any commercial or financial relationships that could be construed as a potential conflict of interest.

## Footnotes

1 https://www.ncbi.nlm.nih.gov/tools/primer-blast/
